# Unexpected Distribution of Chitin and *Chitin Synthase* across Soft-Bodied Cnidarians

**DOI:** 10.3390/biom13050777

**Published:** 2023-04-29

**Authors:** Lauren E. Vandepas, Michael G. Tassia, Kenneth M. Halanych, Chris T. Amemiya

**Affiliations:** 1 Benaroya Research Institute at Virginia Mason, Seattle, WA 98101, USA; 2 Department of Biology, University of Washington, Seattle, WA 98195, USA; 3 Department of Biological Sciences, Auburn University, Auburn, AL 36849, USA; 4 Department of Biology, Johns Hopkins University, Baltimore, MD 21218, USA; 5 Departments of Biology and Marine Biology, University of North Carolina Wilmington, Wilmington, NC 28403, USA; 6 Department of Molecular and Cell Biology, University of California at Merced, Merced, CA 95343, USA

**Keywords:** *chitin synthase*, cnidaria, *Nematostella vectensis*, mesoglea, soft chitin

## Abstract

Cnidarians are commonly recognized as sea jellies, corals, or complex colonies such as the Portuguese man-of-war. While some cnidarians possess rigid internal calcareous skeletons (e.g., corals), many are soft-bodied. Intriguingly, genes coding for the chitin-biosynthetic enzyme, *chitin synthase* (CHS)*,* were recently identified in the model anemone *Nematostella vectensis,* a species lacking hard structures. Here we report the prevalence and diversity of CHS across Cnidaria and show that cnidarian *chitin synthase* genes display diverse protein domain organizations. We found that *CHS* is expressed in cnidarian species and/or developmental stages with no reported chitinous or rigid morphological structures. Chitin affinity histochemistry indicates that chitin is present in soft tissues of some scyphozoan and hydrozoan medusae. To further elucidate the biology of chitin in cnidarian soft tissues, we focused on *CHS* expression in *N. vectensis*. Spatial expression data show that three *CHS* orthologs are differentially expressed in *Nematostella* embryos and larvae during development, suggesting that chitin has an integral role in the biology of this species. Understanding how a non-bilaterian lineage such as Cnidaria employs chitin may provide new insight into hitherto unknown functions of polysaccharides in animals, as well as their role in the evolution of biological novelty.

## 1. Introduction

Chitin, an unbranched long-chain glycopolymer, is the second-most prevalent biomolecule on earth and is present across a wide array of eukaryotic taxa [1,2,3]. Though widely occurring throughout Metazoa, chitinous structures have been studied most thoroughly in arthropod cuticles [4], mollusk radula [5], and annelid chaetae [6]. Recently, chitin, long assumed to be absent from vertebrates, has been shown to be endogenously produced in fishes and amphibians [7,8]. Intriguingly, some gastropod mollusk epidermal chitin and vertebrate chitin are present outside of hard skeletal tissues (e.g., as a component of the gel-like substance in ampullae of Lorenzini in skates) [8,9,10], demonstrating that this glycopolymer is utilized in more diverse contexts than previously realized. 

The chitin biosynthetic pathway is present across Opisthokont clades [11,12], though it appears to be absent in some non-bilaterian metazoan lineages ([2]; this study). The terminal chitin-assembling enzyme, *chitin synthase*, is normally localized to plasma membranes, where several transmembrane domains create a pore through which the newly synthesized chitin molecule is secreted extracellularly [13]. The enzymatically active glycosyl transferase domain is the defining motif of the *chitin synthase* enzyme, and the protein sequence of this domain is highly conserved [2,12,13,14]. 

Metazoan *chitin synthases* fall into two subfamilies, CHS Type I and CHS Type II, where each subfamily can be defined by its unique domain architecture, and phylogenetic analyses have resolved them as reciprocally monophyletic [2]. CHS Type I enzymes contain sterile alpha motifs (SAMs), a domain known to be involved in a diversity of protein–protein, membrane lipid, and RNA interactions [15,16]. However, the function of SAM domains in animal *chitin synthase* enzymes has not been well described. All Type II *chitin synthases* lack SAMs, and some orthologs contain myosin motor domains [17]. Chitin utilization in animals has been most thoroughly investigated in rigid anatomical structures; however, recent reports of *chitin synthase* (*CHS*) genes in cnidarian species that do not possess rigid exo- or endoskeletons suggest that chitin may be being used in more taxa and in different contexts than previously realized [2,8].

**Figure 1:**
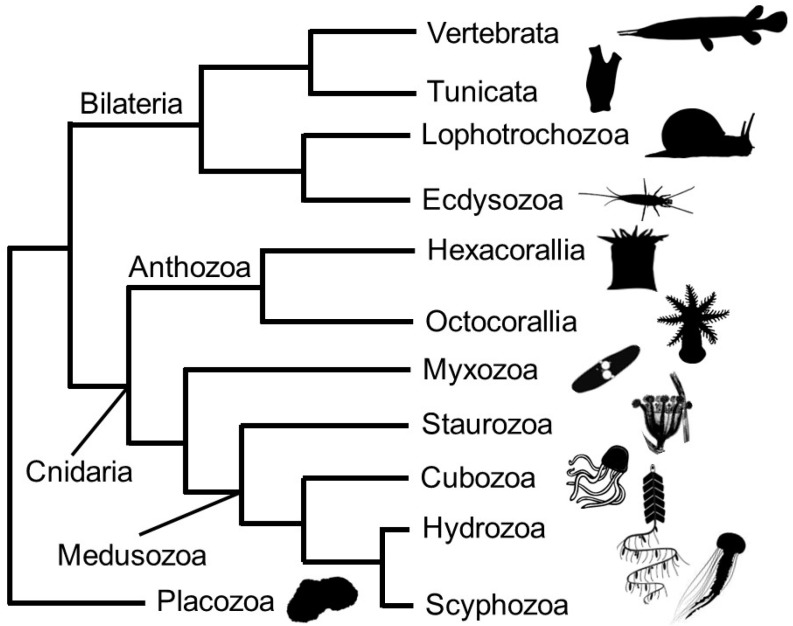
Relationships of Cnidaria (after Zapata et al. 2015; [26]). Cnidarians are the sister taxon to Bilateria, which consists of deuterostomes (chordates, echinoderms, and hemichordates) and protostomes (ecdysozoans, lophotrochozoans). Anthozoans (corals, anemones) are the earliest-diverging extant cnidarian clade. The Medusozoa clade is comprised of lineages that have medusa stages, though not all species do. Myxozoans are a parasitic clade with highly derived body plans but still possess cnidae. Animal silhouettes are available from PhyloPic (phylopic.org).

Cnidarians are the sister clade to Bilateria [18,19,20], and their phylogenetic placement is key for understanding both bilaterian origins and the evolution of metazoans. Cnidarians exhibit diverse life histories and body plans—from individual polyps to large colonies to free-swimming medusae—making this group an intriguing and complex system for studying the emergence of novel structures (Figure 1) [21,22]. Some cnidarian taxa are soft-bodied or “gelatinous” (e.g., the medusae of scyphozoans and hydrozoans—“jellies”); however, while chitinous structures have been described in several discrete lineages within Cnidaria (e.g., stolons of colonial hydrozoans, black coral endoskeletons) [23,24,25], the occurrence of chitin throughout Cnidaria is largely undescribed.

Intriguingly, putative *CHS* genes were identified in the model anthozoan cnidarian, *Nematostella vectensis* [2], which lacks any structures that are obviously rigid or chitinous. The distribution of *CHS* genes throughout Cnidaria has not been explored. Leveraging the recent availability of transcriptomic and genomic resources for diverse cnidarian species, we report *CHS* homologs across Cnidaria and use phylogenetic inference to show that species within almost all major cnidarian clades possess *CHS* genes. We assessed the diversity and gene genealogy of *chitin synthases* across Cnidaria and confirmed the presence of chitin in cnidarian soft tissues using affinity histochemistry. We observed chitin in tissues of multiple cnidarian species. Further, we show that each *Nematostella CHS* ortholog has a discrete expression pattern throughout development. Our findings suggest that “non-rigid chitin” is functionally deployed in cnidarians.

## 2. Materials and Methods

### 2.1. Animal Collection and Sample Preparation

Hydrozoan (*Catablema nodulosa*; *Aequorea victoria*) and scyphozoan (*Phacellophora camtschatica*) medusae used for chitin histology were collected from Puget Sound, WA (47°40′39” N, 122°24′39” W) public docks using a dipper (plastic beaker attached to PVC piping). *Nematostella vectensis* were collected from the Duwamish Waterway (Herring House Park) with a collection permit from WA Department of Fish and Wildlife. *Hydra* were generously supplied by Celina Juliano and maintained as previously described [27]. Wild-caught hydrozoan and scyphozoan species were maintained in 32 ppt seawater (Instant Ocean) at 6 °C. All animal samples were not given food for 24 h prior to fixation to clear gut contents and were thoroughly rinsed in seawater prior to fixation. Medusae and hydroid polyps were fixed either in 4% paraformaldehyde (PFA) or Lavdovski’s fixative (ethanol:formaldehyde:acetic acid:ddH_2_O; 50:10:4:36) for 1 h at 4 °C or 16 h at 4 °C, respectively. *Nematostella* were maintained and fixed as described previously [28].

### 2.2. Description of Chitin-Binding Domain Peptide Probe

To detect the presence and distribution of chitin, we utilized a fluorescent-tagged probe that includes a chitin-binding domain (CBD) from a chitinase of the bacteria *Bacillus circulans*. This peptide probe has been employed to detect and label chitin in an array of animal taxa, including squid, insects, and fishes [7,29,30,31]. A complete preparation protocol for the fluorescent chitin-binding probe is described in [7].

### 2.3. Tissue Embedding and Sectioning

Fixed cnidarian tissues were equilibrated in 15% sucrose in phosphate-buffered saline (PBS) for three hours at room temperature and then in 15% sucrose/7.5% gelatin in PBS at 37 °C for three hours. Samples were then infiltrated with 20% gelatin in PBS overnight at 37 °C and embedded in fresh 20% gelatin in PBS using plastic molds. Embedded samples were mounted onto a cryotome chuck with Tissue-Tek O.C.T. compound (VWR) and frozen in liquid nitrogen. Embedded samples were sectioned at ~7 μm on a Cryostat cryotome and mounted on charged Superfrost Plus slides (VWR).

### 2.4. Chitinase Treatment

Chitin was digested utilizing a chitinase enzyme isolated from the nematode *Brugia malayi* (New England Biolabs, Ipswitch, MA, USA). Samples were permeabilized with PBS + 0.2% Triton X-100 and equilibrated for 1 h at room temperature in chitinase buffer (20 mM Na_2_HPO_4_ pH 6.0, 200 mM NaCl, 1 mM EDTA, 500 μg/mL BSA). Samples were incubated at 37 °C for approximately 16 h in a 1:20 chitinase solution and thoroughly washed in PBS prior to subsequent CBD affinity histochemistry. Fluorescent signals for CBD probe binding between chitinase-treated and buffer control samples were calculated in ImageJ (National Institutes of Health, Bethesda, MD, USA).

### 2.5. Chitin Affinity Histochemistry

Cnidarian tissues were permeabilized with 0.5% Triton X-100 in PBS. Tissue sections were de-gelatinized with 0.3% gelatin in 50% ethanol PBS for 15–30 min at room temperature in a glass slide holder. Slides were then washed for 5 min in PBS and rinsed briefly in water. Slides were allowed to air dry completely prior to staining. Sections were permeabilized in 0.2% Triton X-100 in PBS. Samples were blocked in Protein-Free T20 (TBS) blocking buffer (Thermo Scientific, Waltham, MA, USA) for one hour at RT or overnight at 4 °C. Slides were then incubated with CBD-546 (1:40) and DAPI (1:1000) (4′,6-diamidino-2-phenylindole; Sigma-Aldrich, St. Louis, MO, USA) in TBS blocking buffer overnight at 4 °C. Samples were thoroughly washed in PBS + 0.1%Tween-20, mounted in VectaShield (Vector Laboratories, Newark, CA, USA), and imaged.

Stereoscope imaging was performed on a Leica M205FA (Leica Microsystems, Richmond, IL, USA) fluorescent stereoscope equipped with a DFC360FX monochrome CCD camera and a DFC425C color CCD camera. Epifluorescent images were taken using a Leica DMR upright epifluorescent microscope equipped with a SPOT RT Slider cooled 1.4-megapixel color/monochrome CCD camera (Diagnostic Instruments, Sterling Heights, MI, USA) and an Insight 4 megapixel color CCD camera (Diagnostic Instruments). Confocal images were obtained with a Leica TCS SP5 laser scanning confocal microscope. Image editing was performed using ImageJ (National Institutes of Health freeware).

### 2.6. Semi-Quantitative PCR, Probe Synthesis, In Situ Hybridization

Total RNA was prepared from 50–100 mg of whole adult *Nematostella* homogenized in TRIzol (Invitrogen, Waltham, MA, USA) utilizing a Bullet Blender (Next Advance, Raymertown, NY, USA) and 0.5 mm stainless steel beads (Next Advance). Resulting total RNA was then isolated and purified using the PureLink RNA Mini kit (Ambion, Naugatuck, CT, USA) and treated with DNase I (Ambion) to eliminate potential genomic DNA contamination. RNA was reverse-transcribed to cDNA using the SuperScript III First-Strand Synthesis System (Invitrogen) to generate cDNA. The cDNA was then used as a template for PCR or semi-quantitative PCR to amplify target genes: Nv-actin XP_001630583 (5′-CTATCCAGGCCGTACTCTCCC3′; 5′TAGTGGAACCACCAGACAAGA-3′), Nemve1|93407 (CHS1) (5′-TTCATGGTGGCTGCACTGAT-3′; 5′-CACTCGGCTCCGTATAGCTG-3′), Nemve1|104030 (CHS2) (5′-GATGCAGTTCAGGTGGCGTC-3′; 5′-GTTGTCACACCGGTAGCGT-3′), Nemve1|123712 (CHS3) (5′-ACCGGAGTTACCCATCCAGA-3′; 5′-GCTGATTTGCCTCGTGCATT-3′). For semi-quantitative PCR, cDNA from each sample was normalized to 1 µg. *Nv-CHS* expression was normalized to *Nv-actin* using ImageJ [32].

Target genes were cloned into the TOPO-PCRii vector (Invitrogen), and RNA probes were synthesized by in vitro transcription (MEGAScript Kit; Ambion) driven by T3 or T7 RNA polymerase with DIG incorporation (Roche). Embryos and larvae used for in situ hybridization or chitin histochemistry were collected at time-points that coincide with major developmental stages (early planula, late planula, tentacle bud, primary polyp). Animals were relaxed in 3% MgCl_2_ in FSW for 15 min. Embryos and larvae were fixed in 4% PFA and 0.2% glutaraldehyde in PTw for 1 min at room temperature, and then in 4% PFA for 1 h at 4 °C on a rotating platform. Embryos were thoroughly washed in PTw, gradually dehydrated into methanol, and stored at −20 °C. *Nematostella vectensis* embryos, larvae, and adults were processed for in situ hybridization as previously described [33,34].

### 2.7. Bioinformatics Pipeline

#### 2.7.1. Identification of CHS Homologs

Full transcriptomic and gene-model cnidarian datasets were acquired from [35], representing 67 cnidarians and 8 outgroup taxa (Supplementary Table S1). Nucleotide sequences were translated using TransDecoder (https://github.com/TransDecoder/TransDecoder accessed on 2 June 2018), and the resulting amino acid sequences were used for all subsequent bioinformatic analyses. To identify proteins with putative function in chitin synthesis, HMMER version 3.1b2 [36] was used to annotate sequences predicted to possess at least one *chitin synthase* domain (CHS2; PF03142; [37]).

Using *hmmsearch,* the CHS2 domain model was searched against each translated transcriptome to identify proteins putatively possessing CHS2 domains meeting *hmmsearch’*s default detection thresholds. Protein sequences predicted to possess CHS2 domains were then isolated from their encompassing datasets and annotated with full domain architecture using *hmmscan* and the Pfam domain database (version 32.0; [38,39]). All sequences containing a best-fit CHS2 domain meeting *hmmscan’s* default inclusion threshold were then isolated. These isolated CHS2-containing sequences were clustered per dataset using *cd-hit* version 4.6 (*-c 0.95;* [40]) to remove redundant proteins due to transcript fragments or isoforms.

#### 2.7.2. Phylogenetic Analysis

Unique cnidarian sequences that possess CHS2 domains (n = 95) were supplemented with 63 additional *chitin synthase* proteins identified on the NCBI sequence repository (Supplemental Table S1) using NP_524209.3 (*chitin synthase* 2, isoform D (*Drosophila melanogaster*)) and analyzed. Multiple sequence alignment containing all 158 sequences was obtained using MAFFT’s L-INS-I algorithm. Best-fit substitution model using Bayesian information criterion was inferred using ModelFinder (-*m MFP* flag; [41]) included in the IQ-TREE 1.6.12 distribution. Following model inference, IQ-TREE was used to infer a maximum-likelihood topology of *chitin synthase* proteins [42] and perform ultrafast bootstrapping for node support [43]. All nodes with <95% ultrafast bootstrap support were collapsed as polytomies.

Using recently available public resources for single-cell gene expression data from *Hydra* (https://singlecell.broadinstitute.org/single_cell/study/SCP260/stem-cell-differentiation-trajectories-in-hydra-resolved-at-single-cell-resolution (accessed on 12 January 2023)) [44], we searched for cellular-level expression distribution of *Hv-CHS* transcripts in adult *Hydra*. A t-SNE plot of shared nearest-neighbor (SNN)-clustered single-cell data was generated for each *CHS* homolog: *HV-CHS1* (t13590aep|CHS3_CRYNH) and *HV-CHS2* (t23128aep|CHS6_USTMA). Sequences for *Hydra CHS* orthologs are detailed in Supplemental Table S1.

## 3. Results

### 3.1. Predicted Homologs for the Enzyme Chitin Synthase (CHS) Are Present in Most Recognized Cnidarian Clades and Expressed in Taxa or Life Stages with No Reported Rigid Structures

**Figure 2:**
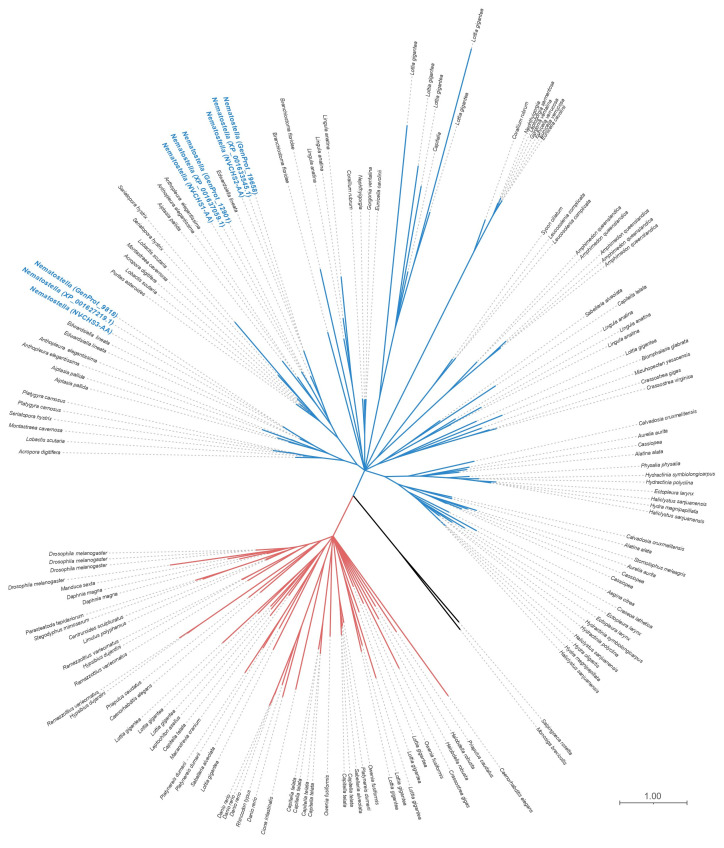
Evolutionary relationships of cnidarian *chitin synthases* inferred by maximum likelihood (ML) phylogenetic influence. Taxa from Anthozoa, Scyphozoa, Hydrozoa, and Staurozoa have *chitin synthase* genes. All resolved cnidarian CHSs fall within the Type II clade (**blue**) in addition to several CHS sequences identified among lophotrochozoans. In contrast, the Type I clade (**red**) comprises sequences identified across Bilateria. Sister to each clade are non-metazoan CHS genes (**black**). Two cnidarian sequences have been pruned from the Type I CHS clade as extreme branch-length outliers. *Nematostella* CHS sequences are highlighted for visualization purposes only. All nodes possess ultrafast bootstrap support ≥95%. For sequence references and species abbreviations, see Table S1.

Using recently available transcriptomic and genomic data representative of deep cnidarian taxon sampling [2,35], we identified *CHS* genes in thirty-two cnidarian species (Figure 2; Supplemental Table S1). We did not identify *CHS* homologs in myxozoans. Cnidarian *chitin synthases* cluster in the metazoan CHS Type II clade, as defined previously [2]. Some cnidarian taxa appear to have undergone lineage-specific expansions of *CHS* genes, particularly in sea anemone *Aiptasia* (Anthozoa–Hexacorallia–Actinaria) and the coral *Montastrea cavernosa* (Anthozoa–Hexacorallia–Scleractinia), which have four and five predicted *chitin synthases* in their genomes, respectively (Supplemental Table S1).

Maximum likelihood phylogenetic inference shows a complex evolutionary history for metazoan *CHS*, where all metazoan *CHS* sequences fall within either of the previously described Type I or Type II CHS clades (Figure 2; [1]). Both metazoan *CHS* clades are individually resolved as sister to non-animal *CHS*. Intriguingly, we did not identify *CHS* homologs in placozoan or ctenophore genomes, suggesting independent losses in those non-bilaterian lineages. Cnidarian taxa that possess more than one predicted *CHS* gene do not always have paralogs that cluster together; instead, these cases reflect shared ancestral diversification rather than crown-group expansions. 

**Figure 3:**
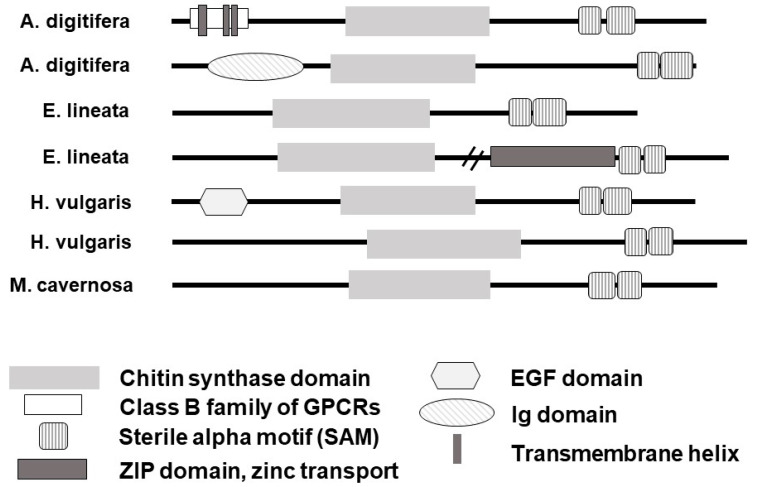
*CHS* domain diversity in Cnidaria. Almost all full-length cnidarian CHS sequences contain at least one protein-binding or RNA-binding sterile alpha motif (SAM) domain. Within taxa, *CHS* genes encode diverse domains, which may indicate specialized functions among *CHS* paralogs. Horizontal dashes indicate removal of non-domain-containing sequences due to space restrictions.

Domain organization of cnidarian *chitin synthase* genes varies (Figure 3). Some cnidarian *chitin synthases* contain EGF domains and protein-binding domains such as sterile alpha motifs (SAMs). A largely complete scleractinian *CHS* sequence from the coral *Acropora digitifera* includes several predicted transmembrane regions, consistent with the expected localization of the *chitin synthase* enzyme to the cell membrane [13].

### 3.2. Chitin Is Present in Cnidarian Soft Tissues

**Figure 4:**
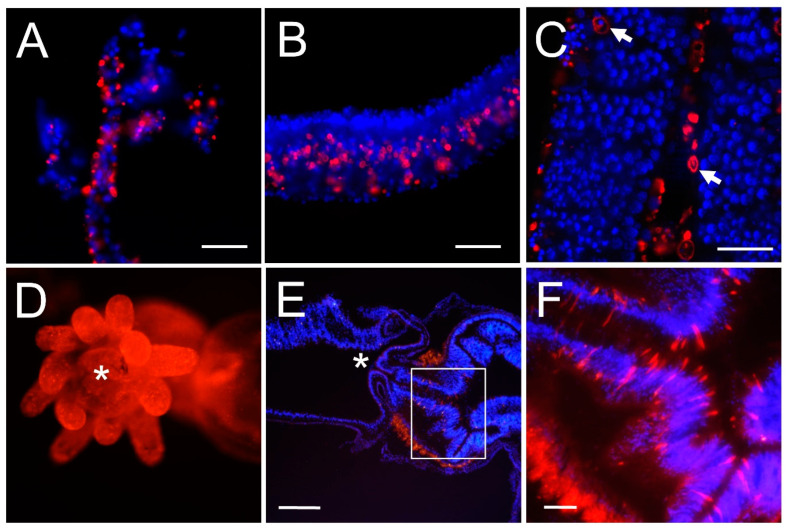
Chitin is found in the adult tissues of scyphozoan and hydrozoan medusae and in an anthozoan. Nuclei (blue, DAPI). Chitin (red, CBD-546). (**A**) Scyphozoan (*Phacellophora camtschatica*) tentacle. Chitin is widely present in the dermis throughout the tentacle. Scale bar: 50 µm. (**B**) Tentacle of hydrozoan medusa *Catablema nodulosa*. Scale bar: 50 µm. (**C**) A cryosection of hydrozoan medusa *Aequorea victoria* bell tissue shows chitin is widely present. Some chitin staining is acellular, though several cells show chitin labeling within them. Arrows point to individual cells where chitin labeling appears to be localized to the plasma membrane. Scale bar: 50 µm. (**D**) Oral view of chitin labeling in whole adult *Nematostella* shows that chitin is widely present. Asterisk marks the location of the mouth. (**E**) Chitin labeling is widely present throughout *Nematostella vectensis* tissue cryosections, including the tentacles, pharynx, body wall, and mesoglea. Mouth is marked with an asterisk. Scale bar: 200 µm. Chitin labeling is widely present throughout Nematostella tissues. Mouth is pointing left. Scale bar—200 µm. (**F**) Magnified view (white box) in (**E**), showing chitin labeling in the pharynx. Individual elongated cells are stained for chitin. Scale bar—50 µm.

To assess whether chitin is present in cnidarian tissues, we performed fluorescence chitin affinity histochemistry on hydrozoan (*Catablema nodulosa*; *Aequorea victoria*) and scyphozoan (*Phacellophora camtschatica*) medusae tissues and on an anthozoan (*Nematostella vectensis*). We found that chitin is broadly distributed in tissues that are not associated with rigid skeletal structures (Figure 4). *Phacellophora camtschatica* tentacle, *Catablema nodulosa* tentacle, and *Aequeorea victoria* bell tissue show chitin labeling (Figure 4A–C). Chitin labeling is present broadly in the anemone *Nematostella vectensis* (Figure 4D–F). The distribution of chitin appears to be largely acellular, consistent with the canonical process of secretion of the chitin molecule from chitin-producing cells into extracellular spaces. Some cells show chitin labeling in the cell periphery (Figure 4C, arrows), possibly corresponding to membranes of cells actively synthesizing chitin.

To confirm that the fluorescent chitin-binding domain (CBD) probe was binding to chitinous structures, whole adult *Nematostella* were incubated with the chitin-digesting enzyme chitinase. Chitinases degrade chitin by breaking glycosidic bonds along the chitin polymer [45]. Chitinase digestion experiments show that the CBD probe binds preferentially to chitin in *Nematostella* (Supplemental Figure S1), as labeling was significantly reduced in enzyme-treated samples by an average of 40% across sample images (Supplemental Figure S1A,C) compared to controls (Supplemental Figure S1B,D).

### 3.3. The Distribution of Chitin and Expression of Chitin Synthases in Hydra

**Figure 5:**
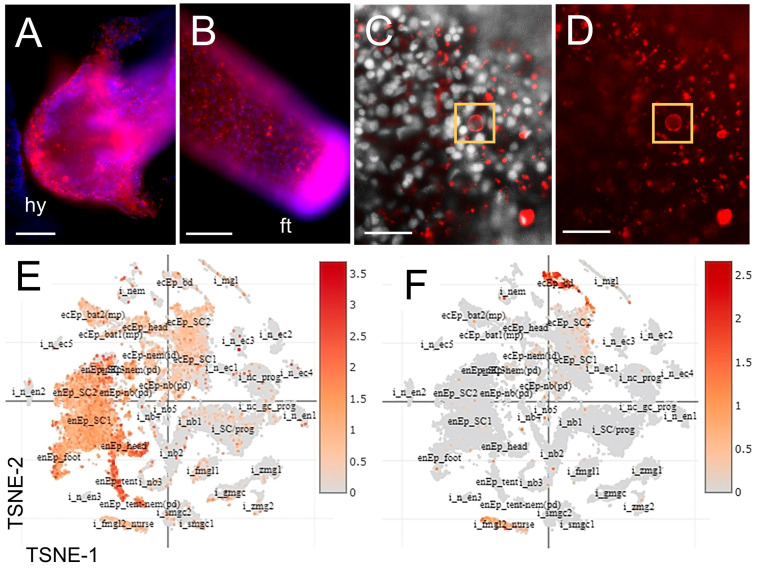
*Hydra* expresses *CHS* in diverse tissues. (**A**) Chitin labeling in whole *Hydra vulgaris*. There is widespread chitin labeling in the hypostome. (**B**) At the aboral pole, the distal-most portion of the foot is strongly labeled for chitin. (**C**) Detail of chitin staining in *Hydra* trunk epidermal tissues. CBD labeling shows broad distribution of chitin in the epidermis. (**D**) The capsules of some nematocysts appear to be chitinous (yellow box). (**E**) Single-cell expression analysis of *HV-CHS1* (t13590aep|CHS3_CRYNH) as visualized with the *Hydra* single-cell transcriptome portal [44] shows expression in endodermal and ectodermal epithelial cells, and in nematocytes. (**F**) Expression of *HV-CHS2* (t23128aep|CHS6_USTMA) as visualized with the *Hydra* single-cell transcriptome portal [44] shows expression in the ectoderm of the basal disc and in female gonadal cells. Nuclei, blue or grey (DAPI); chitin labeling is red (CBD-546). Hy—hypostome (oral region); Tn—tentacle. (**A**,**B**) scale bar—100 μm. (**C**,**D**) scale bar—50 μm.

Fluorescent histochemical labeling of chitin in *Hydra* shows that chitin is prevalent in the head (Figure 5A), with especially intense chitin labeling in the foot (Figure 5B). The *Hydra* foot is the structure that attaches the polyp to the substrate, and other hydrozoan polyp species have been shown to have a prevalence of chitin stabilizing the stolons [24,46]. *Hydra* trunk tissue shows punctate chitin labeling (Figure 5C,D; Supplemental Figure S2), with individual cells that appear to have chitin localized to the cell membrane (arrows).

The model solitary hydrozoan polyp *Hydra vulgaris* has two predicted *chitin synthase* orthologs (*Hm-CHS1*: XP_012554922.1, t13590aep; *Hm-CHS2*: XP_004207525.2, t23128aep) [2,44]. Single-cell gene expression data from *Hydra* [44] show that *HmCHS-1* is broadly expressed throughout the animal in both endodermal and ectodermal epithelial cells, and in nematocytes (Figure 5E). *Hm-CHS1* is most highly expressed in tentacle epithelial cells derived from endoderm, nematoblasts, and endodermal epithelial cells in the head. *Hm-CHS2* expression is more restricted and is localized primarily to the ectoderm of the basal disc and in female gonadal cells (Figure 5F).

### 3.4. Chitin Synthase Genes Are Differentially Expressed in the Model Sea Anemone Nematostella vectensis during Development

*Nematostella vectensis* is an established cnidarian model for studying metazoan evolution and developmental processes [47]. Embryonic and larval development in *Nematostella* has been well documented [48,49,50]. In brief, approximately 24 h post-fertilization (24 hpf), the *Nematostella* gastrula organizes into a ciliated, free-swimming planula larva with an apical sensory tuft at the aboral pole. As development progresses, mesenteries—multifunctional tissues comprising muscles, digestive cells, and gonads—form via continued inward migration of endoderm and ectoderm. Tentacle tissue organizes at the oral pole of the planula, and four projections of tissue—tentacle buds—form around the site of the mouth. The tentacle buds and body column gradually elongate, and the planula settles onto the substrate and metamorphoses into a polyp. Primary polyps initially possess four tentacles and two mesenteries.

#### 3.4.1. Three Nematostella CHS Paralogs Are Expressed during Development

**Figure 6:**
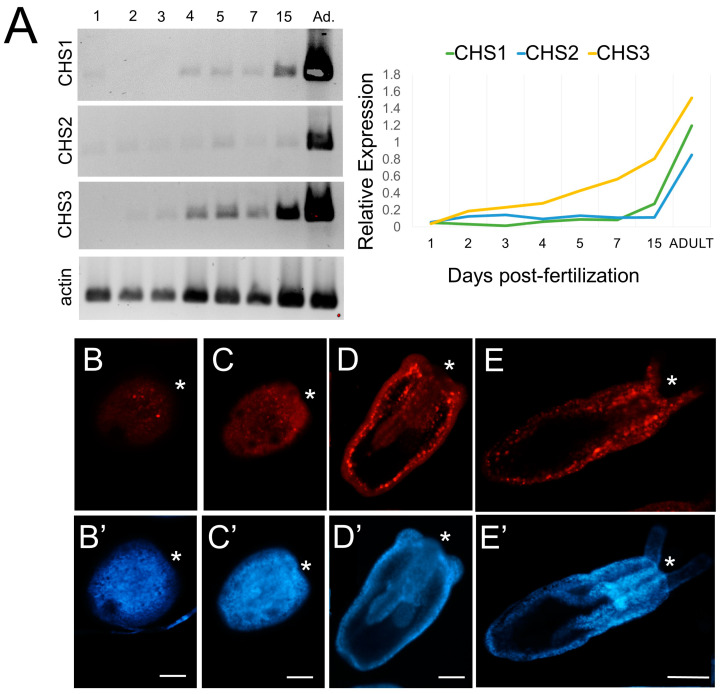
Three *chitin synthase* paralogs are expressed in *Nematostella vectensis* during development. (**A**) Electrophoresis gels (left) showing semi-quantitative PCR of *Nv-CHS* through development (1 dpf through 15 dpf). Concentrations of cDNA were normalized to 1 µg. Normalized semi-quantitative PCR expression of three predicted *chitin synthase* genes during *Nematostella vectensis* development (right). (**B**–**E**) Chitin histochemistry in wild-type *Nematostella* development. Confocal images of chitin labeling at sequential developmental stages. Scattered cells labeled with chitin in early larval stages (**B,B’**,**C,C’**). In the tentacle bud stage (**D**,**D**’) chitin labeling is abundant along the body column and beneath the budding tentacles (arrows). Chitin is widely distributed in the primary polyp stage (**E**,**E**’). Oral end labeled with an asterisk (*). Day post-fertilization (dpf); Adu.—adult polyps. All panels—chitin (red, chitin-binding domain CBD-546 probe), nuclei (blue, Hoechst). Scale bar—50 µm in all panels.

*Nematostella* has three *CHS* paralogs in its genome ([51]; this study). Semi-quantitative PCR shows that transcript abundance for all three *CHS* genes increases through development, with the highest expression levels being in the primary polyp (15 days post-fertilization) and adult (Figure 6A). **chitin synthase*-1* (Nemve1|93407) is faintly detectable at 24 hpf, and expression increases at 4 dpf through the adult stage. **chitin synthase*-2* (Nemve1|104030) expression is detectable at 24 hpf and gradually increases; this gene appears to have the lowest relative expression of the three CHS orthologs. **chitin synthase*-3* (Nemve1|123712) expression is detectable at 48 hpf, with strong expression through the rest of development and in the adult. The expression levels of *chitin synthase* genes were normalized to actin expression (Figure 6A, bottom panel).

#### 3.4.2. Chitin Is Distributed throughout the Developing Planula and Primary Polyp

In *Nematostella* planula stages, the CBD probe labels extracellular areas and scattered cells (Figure 6B–E). Chitin labeling is prevalent in the body column and in the pharynx of the tentacle bud larva (Figure 6D). There is concentrated chitin labeling under the budding tentacles (Figure 6D, arrows). In primary polyps, there is widespread chitin labeling (Figure 6D) that is similar to chitin labeling in adult *Nematostella* tissues (compare to Figure 4D–F). Chitin is not detectable by histochemistry prior to the late planula stage (approximately 144 hpf; Figure 6B), possibly due to the reduced sensitivity of the CBD probe compared to other detection methods we have applied to assay chitin synthesis (e.g., RT PCR, in situ hybridization).

#### 3.4.3. *Chitin Synthases* Are Differentially Expressed in the Ectoderm during Nematostella Development

**Figure 7:**
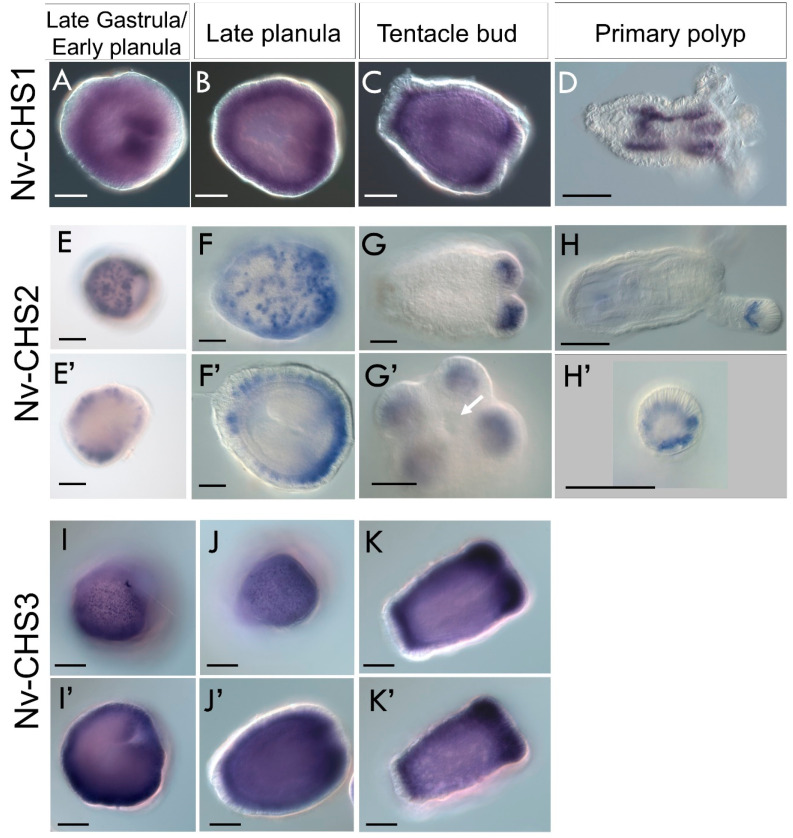
*Nematostella vectensis *chitin synthases** are expressed in the ectoderm during development. Typical expression patterns of Nv-*CHS* genes in late gastrula, planula, tentacle bud, and primary polyp stages. Oral end facing right in all panels. (**A**–**D**) *Nv-CHS1* is expressed diffusely throughout the ectoderm, with highest levels of expression in the pharyngeal ectoderm. (**B**) In the late planula stage, *CHS-1* is expressed throughout the ectoderm. (**C**) Expression in the tentacle bud stage occurs throughout the ectoderm but is highest at the aboral end and in the developing tentacles. (**D**) In the primary polyp, expression is concentrated in the mesenteries. (**E**) *Nv-CHS2* is expressed in a punctate pattern throughout the ectoderm in gastrula/planula stages, becoming concentrated in the oral pole (**F**). (**G**) In tentacle bud stage, expression is concentrated in the developing tentacle ectoderm. (**G**’) View of oral pole of tentacle bud stage, showing nv-CHS2 expression in the developing tentacles. Arrow pointing to mouth. (**H**) In primary polyps, *Nv-CHS2* expression is localized to the tips of the tentacles. (**I**,**J**) *Nv-CHS3* is widely expressed in a punctate pattern throughout the planula body wall ectoderm, and tentacle buds. (**K**) E–K—superficial plane views of samples. (**E’**–**K’**) Deep plane views of larvae. Scale bar—50 µm in all panels.

To assess the tissue-level distribution of *chitin synthase* expression, we performed in situ hybridization on developing *Nematostella*. All three *Nv-CHS* genes are expressed in the ectoderm, with distinct expression patterns in the ectoderm of the late gastrula, planula, tentacle bud, and primary polyp stages (Figure 7). *Nv-CHS1* is expressed diffusely throughout the embryo ectoderm and is most highly expressed in the developing pharynx and mesenteries (Figure 7A). In the late planula stage, *Nv-CHS1* is expressed in the body wall, becoming concentrated in the aboral end and in the developing tentacles in the tentacle bud stage (Figure 7B,C). In the primary polyp, expression is localized to the mesenteries (Figure 7D).

*Nv-CHS2* is expressed in a punctate pattern throughout the ectoderm in the gastrula/planula stages, becoming concentrated in the oral pole as development progresses (Figure 7E–H). In the tentacle bud stage, expression is concentrated in the developing tentacle ectoderm (Figure 7G). In primary polyps, expression is localized to the tips of the tentacles (Figure 7H). *Nv-CHS3* is widely expressed in a punctate pattern throughout the planula body wall ectoderm and developing pharynx (Figure 7I,J). In the tentacle bud stage, there is expression throughout the body wall ectoderm with relatively high expression in the tentacle buds and at the aboral pole (Figure 7K). Recently available single-cell sequencing data from *Nematostella* show *Nv-CHS1* (sequence identifier NVE14301) expression mostly in ectodermal cells, while *Nv-CHS3* (sequence identifier NVE8515) is widely expressed in ectodermal cells, and in the gastrodermis [52,53]. The transcript recovered from the Steger et al. single-cell dataset for *Nv-CHS2* (NVE22726) is truncated and unavailable for single-cell expression analysis. The widespread expression of *chitin synthase* genes in *Nematostella* is intriguing, as the animal lacks hard structures that may be obviously chitinous.

## 4. Discussion

Numerous cnidarians with no previous descriptions of chitinous structures possess *chitin synthase* genes. Histochemistry confirms the presence of chitin in the soft tissues of scyphozoan and hydrozoan medusae, as well as in the model species *Hydra* and *Nematostella*. The diversity of domains in cnidarian *chitin synthase* genes suggests that *CHS* paralogs may have specialized to perform multiple functions. The expression of *chitin synthase* and detection of chitin in cnidarian soft tissues suggests an expanded role for chitin in cnidarians.

### 4.1. The Molecular Toolkit for Chitin Synthesis Is Present in More Cnidarian Taxa Than Previously Reported, including in Soft-Bodied Species or Life-History Stages

Chitinous structures have been described in some cnidarian taxa as a component of endoskeletons (e.g., antipatharian anthozoans) or protective coatings (e.g., hydrozoan polyp theca) or comprising morphological stabilizing structures that interface with substrates. For example, some sea anemones (Anthozoa–Hexacorallia) are reported to synthesize a chitinous coating on the basal disc [54]. For many cnidarians, however, collagenous mesoglea or hydrostatic support are common sources of structural form and stability.

Here we report that most major cnidarian clades possess and/or express at least one *chitin synthase* in their genomes or transcriptomes. Transcriptomic data show that several cnidarian lineages that have no previous reports of chitin in their tissues (e.g., Octocorallia, Cubozoa) express *CHS* genes. Chitin has been explicitly described as being absent from octocoral tissues [25]; however, all Octocorallia species assayed in this study possess at least one *CHS* gene. Early works describing the presence of chitin in Scleractinia hypothesized that the zooxanthellae—symbiotic dinoflagellate algae—were the source of chitin in stony coral tissue, allowing for the deposition of the endoskeleton [55]. We show that stony coral species queried in this study possess the genes required to synthesize chitin endogenously. Additional cnidarian species may possess *chitin synthase* genes in their genomes, but these genes are not expressed in the tissues or life stages from currently available transcriptomes and genomic data are not yet available.

### 4.2. Distribution of Chitin in Cnidarian Tissues

The presence of chitin in the stolons and theca of hydrozoan colonies has been documented [54,56,57]. The description of chitin in Scyphozoa has been in the suborder Dactyliophorae and Semaeostomae, where it encapsulates nutrient reserves in strobila or polyps [58,59]. Chitin has not been previously reported within the tissues or structures of any adult scyphozoan or hydrozoan medusae. Using a sensitive affinity histochemistry assay, we show that chitin is present in tissues of adult scyphozoan and hydrozoan medusae. Furthermore, transcriptomic data confirm that genes for chitin synthesis are expressed in soft-bodied adults. Future works should explore how chitin is being deployed in soft tissues, with what proteins or minerals it may be complexed, and which cnidarian cell types are synthesizing chitin.

### 4.3. CHS Is Expressed during Nematostella vectensis Development

We show that all three *chitin synthase* paralogs are expressed in developing *Nematostella,* each with its own pattern of expression. It is unclear which specific cell types are expressing *CHS* and subsequently producing chitin in the soft-bodied anemone *Nematostella*. The punctate expression patterns of *CHS-2* are similar to previously described spatial expression of secretory, neural, or cnidocyte genes [60,61,62,63]. Intriguingly, *Nv-CHS1* and *Nv-CHS3* are expressed abundantly at the aboral pole; chitinous parisarcs of some hydroids and anemone basal discs stabilize the animal’s aboral tissues in its interactions with sediment substrates [54,57].

## 5. Conclusions

Chitin is widely distributed across Cnidaria and is present in soft tissues in multiple cnidarian species. Moreover, the presence of the molecular machinery for chitin assembly in nearly all major cnidarian taxonomic clades and the existence of endogenous chitin in the soft tissues of multiple lineages suggests an important and unexpected role for chitin in cnidarian biology.

We posit that chitin can be deployed in structurally malleable tissues in cnidarians and that it can be complexed with a number of biological compounds (e.g., proteins or other glycomolecules) to achieve a diversity of structural presentations that do not involve firm fibrous chitin strands or mineralization. Cnidarians use chitin in a variety of morphological contexts. All cnidarian clades surveyed, except for Myxozoa, appear to possess the molecular capacity to synthesize chitin, as assessed by their possession of cognate *chitin synthase* homologs. Many cnidarian taxa express multiple paralogs of *chitin synthase*, indicating diverse and integral roles for chitin in their biological processes. Future expression studies and functional analyses will be necessary to determine the nature and precise roles of chitin in soft-bodied cnidarians.

## Data Availability

The sequencing data presented in this study are available in the publicly archived datasets referenced herein.

## Supplementary Materials

The following are available online at https://www.mdpi.com/article/10.3390/biom13050777/s1. Supplemental Figure S1: Stereoscope images of chitin depletion via chitinase digestion in adult *Nematostella*. Nuclei, blue (DAPI); chitin labeling is red (CBD-546). Arrows point to the location of the mouth. Compare fluorescent signal in A vs. B, and C vs. D. Paired figures are labeled nuclei of samples (DAPI). Samples in A and C were treated with chitinase enzyme to digest chitin present in the samples. Samples in B and D were incubated in buffer alone, without enzyme. (A) Oral end (mouth, pharynx, tentacles) of *Nematostella* incubated with chitinase enzyme. Chitin labeling is depleted. (B) Oral end of untreated *Nematostella* showing preserved chitin labeling. (C) Magnified tentacle of specimen digested with chitinase. Chitin histochemical labeling is depleted. (D) Magnified tentacle of untreated specimen showing chitin labeling. Supplemental Figure S2: Detail of chitin staining in Hydra vulgaris trunk epidermis. Nuclei, blue (DAPI); chitin labeling is red (CBD-546). Detail of chitin staining in Hydra vulgaris trunk epidermal tissues. Much of the chitin labeling appears to be acellular, though there are some cells (white arrows) that show chitin labeling within them. It is possible that these cells are synthesizing chitin, given the staining pattern. Chitin is usually assembled near the cell membrane and subsequently secreted. Supplemental Figure S3: Sense probe controls of Nematostella in situ hybridization. Negative controls (sense RNA probes) in Nematostella CHS in situ hybridization. Little background staining is observed. Scale bar—50 µm in all panels. Table S1: Taxonomic and source tissue data for metazoan and choanoflagellate *CHS* sequences [64,65,66,67,68,69,70,71,72,73,74,75,76,77,78,79].
