# Supplementary figures and images for: Unexpected Distribution of Chitin and Chitin Synthase across Soft-Bodied Cnidarians
Source: Biomolecules. 2023 Apr 29;13(5):777. doi: 10.3390/biom13050777 (PMC10216261; doi:10.3390/biom13050777)

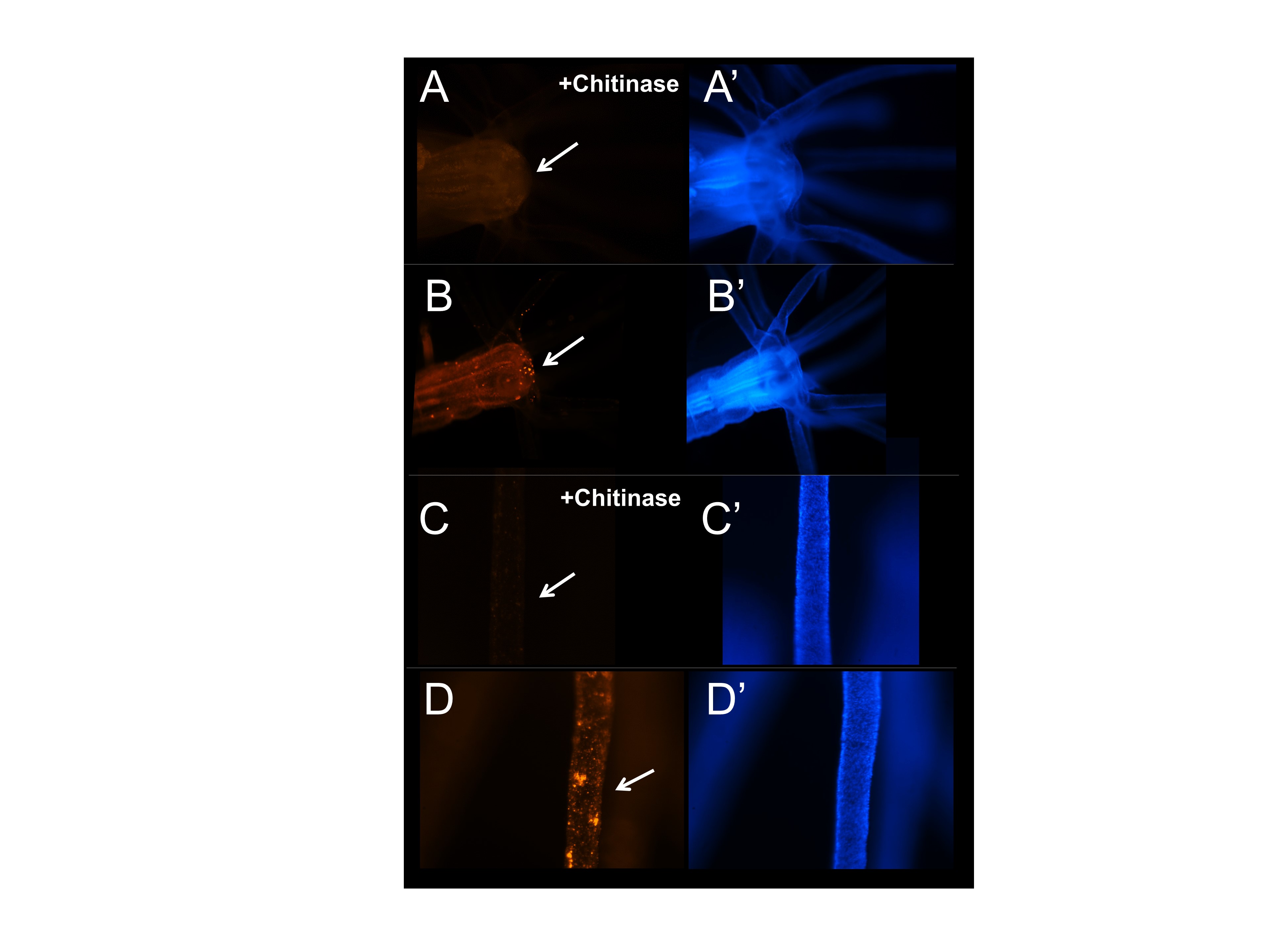

Supplement: Supplementary file 1 [file biomolecules-13-00777-s001.zip › SUPPLEMENTAL FIGURE S1 CHITINASE NEMATOSTELLA.jpg]

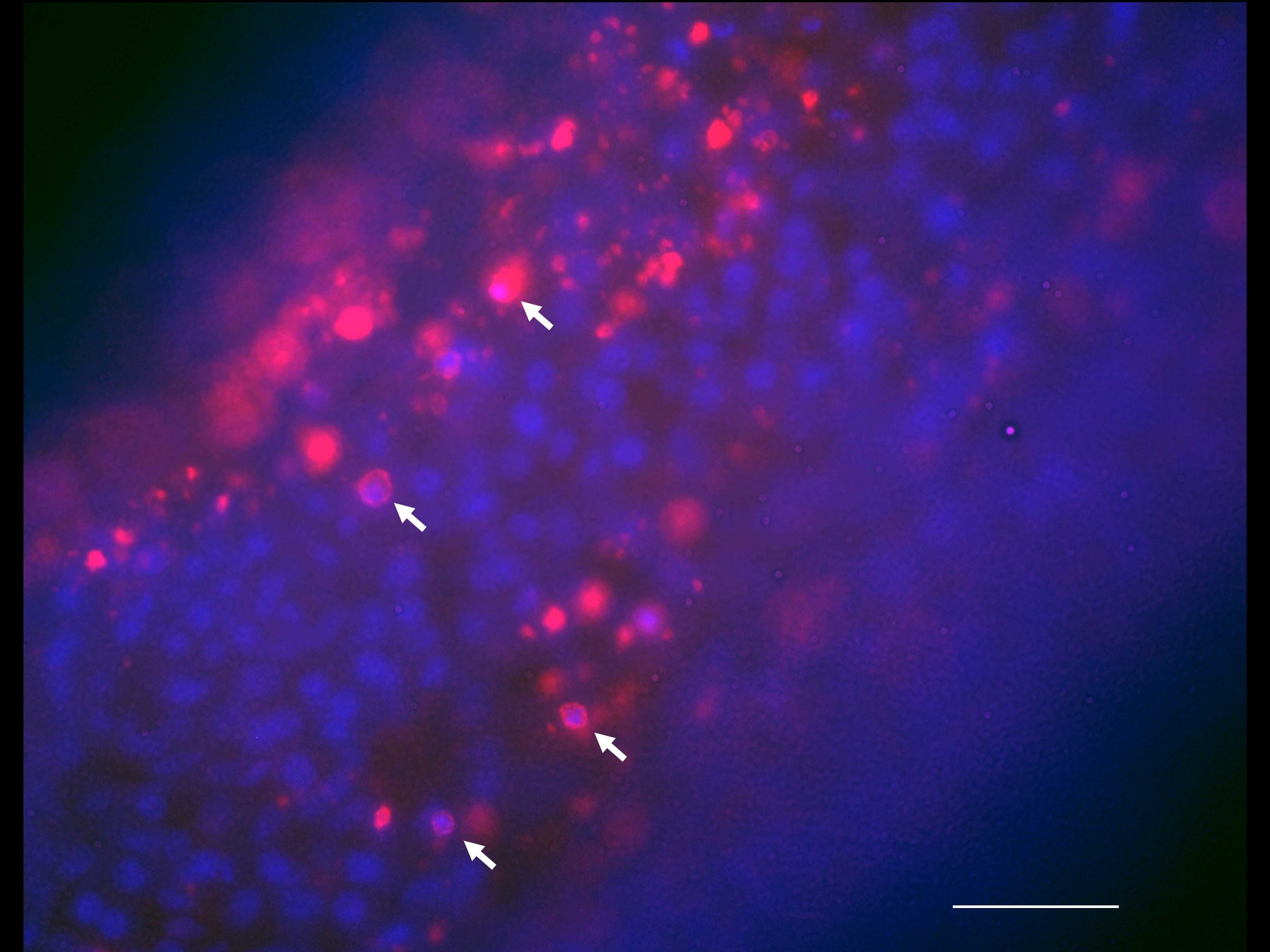

Supplement: Supplementary file 1 [file biomolecules-13-00777-s001.zip › SUPPLEMENTAl FIGURE S2 CHITIN IN HYDRA.jpg]

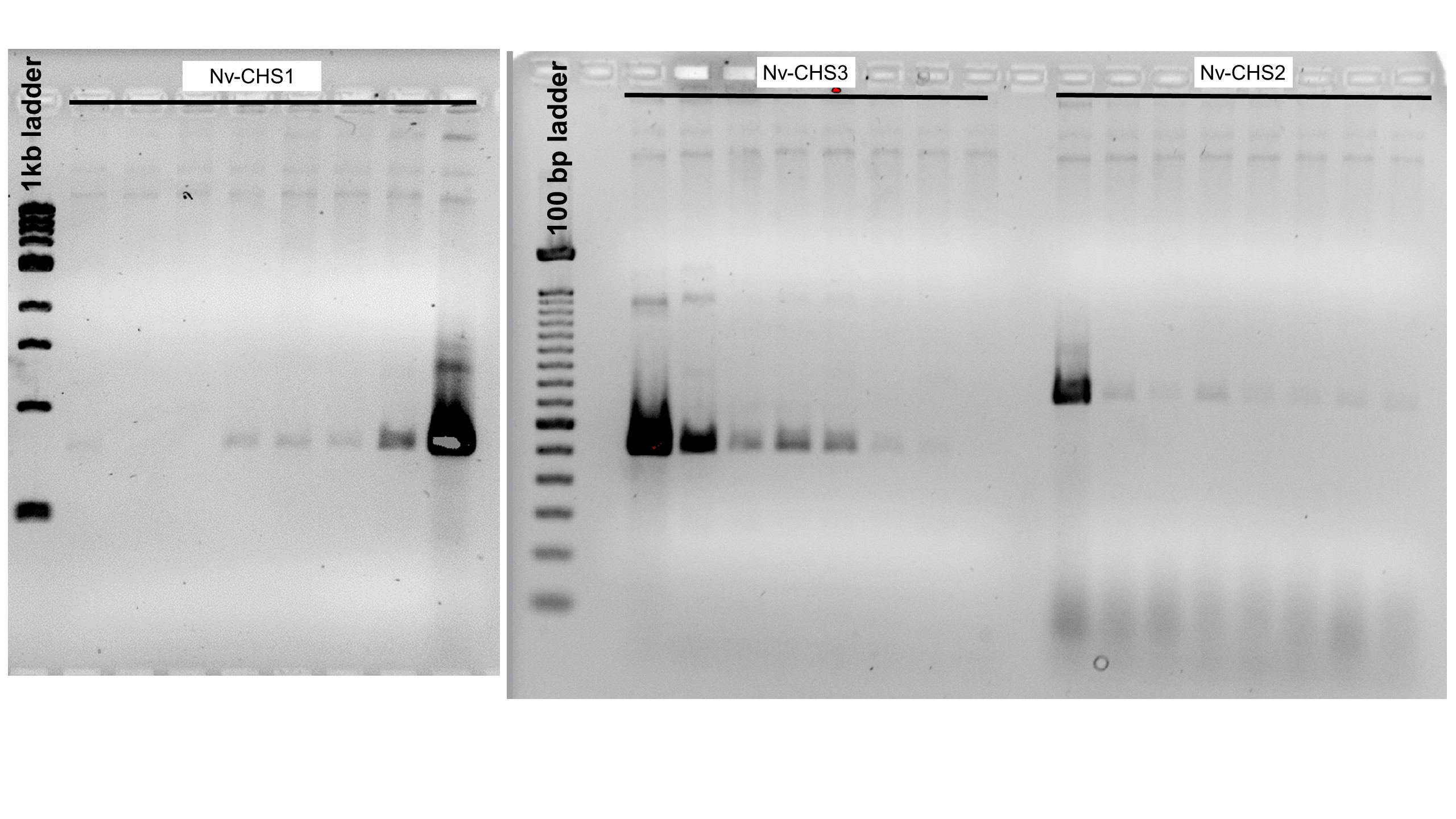

Supplement: Supplementary file 1 [file biomolecules-13-00777-s001.zip › SUPPLEMENTAL FIGURE S3 RT PCR DEVELOPMENT CHS FULL GEL IMAGES.jpg]
